# Late puerperal hemorrhage of a patient with Klippel–Trenaunay syndrome: A case report

**DOI:** 10.1097/MD.0000000000018378

**Published:** 2019-12-16

**Authors:** Jian Zhang, Kana Wang, Jie Mei

**Affiliations:** a Department of Gynecology and Obstetrics, Sichuan Academy of Medical Sciences and Sichuan Provincial People's Hospital; b Department of Gynecology and Obstetrics, West China Second University Hospital and Sichuan University, Chengdu, Sichuan, China.

**Keywords:** Klippel–Trenaunay syndrome, late puerperal haemorrhage, pregnancy

## Abstract

**Introduction:**

The Klippel–Trenaunay syndrome (KTS) is a rare congenital disorder. The obstetric course of women with KTS varies. Complications include bleeding, disseminated intravascular coagulation (DIC), thromboembolic events, etc.

**Patient concerns:**

Here, we report a case of late puerperal hemorrhage of a Chinese puerpera with KTS. The repeating severe hemorrhage, the DIC, and the Kasabach–Merrit syndrome made the treatment more difficult.

**Diagnosis:**

KTS is a mixed malformation with a vascular component that is characterized by abnormal development of veins, capillaries, and lymphatics. Our patient was first diagnosed with KTS at the last trimester of pregnancy.

**Interventions:**

Massive infusion of blood products, two laparotomies, as well as bilateral internal iliac artery embolization was carried out.

**Outcomes:**

Although the patient survived from the life-threatening hemorrhage, she lost her uterus forever.

**Conclusion:**

An interdisciplinary cooperation of obstetrician, anesthesiologist, vascular surgeon, and intensive care physician is highly recommended. Prophylactic anticoagulation is generally advised in the gestational and postpartum period.

## 1 Introduction

The Klippel–Trenaunay syndrome (KTS) is a rare congenital disorder which consists of a triad of cutaneous port-wine capillary malformations, varicose veins and hemihypertrophy of the soft tissues and bone.^[1]^ The maternal and fetal risks associated with pregnancy can be exacerbated in women with KTS. Here, we present a case of late puerperal hemorrhage of a Chinese puerpera with KTS.

The report complies with the Declaration of Helsinki and it was approved by the institutional ethical committee of Sichuan Academy of Medical Sciences and Sichuan Provincial People's Hospital. We were given informed consent from the patient to use the medical history and images.

## 2 Case report

A 26-year-old Chinese Han female patient with a sudden onset of massive vaginal bleeding was transferred to our department by the emergency service. She had delivered a healthy female baby at 39 weeks of gestation by primary cesarean due to the large vulvovaginal varicosities in the local hospital 10 days before. And the referral letter of the regional hospital wrote that there had been postpartum hemorrhage of about 1500 mL (general anesthesia, without telling any other details of the operation). By blood transfusion and the use of uterine contraction medicines, the patient had recovered well and been discharged with her newborn. On the 10th day after delivery when she was at home, there was a sudden heavy vaginal bleeding as mentioned above. On the way to referral, there had been ∼1500 mL of vaginal bleeding. The regional hospital gave her three units of red cell suspension and some drugs to promote uterine contraction and then transferred her to our hospital by emergency.

When she was a little girl, she limped and her left leg was sweaty. She was diagnosed as “acromegaly” in her childhood without any special treatment. She conceived spontaneously without preconception counseling. She was first diagnosed with KTS by one of the dermatologists of the local hospital at the last trimester of pregnancy. There had been no history of thrombosis. During pregnancy, neither low-weight heparin nor low-dose acetylsalicylic acid had been given to her. Her medical history was otherwise uneventful. She denied family history of KTS or any other familial genetic diseases.

**Figure 1:**
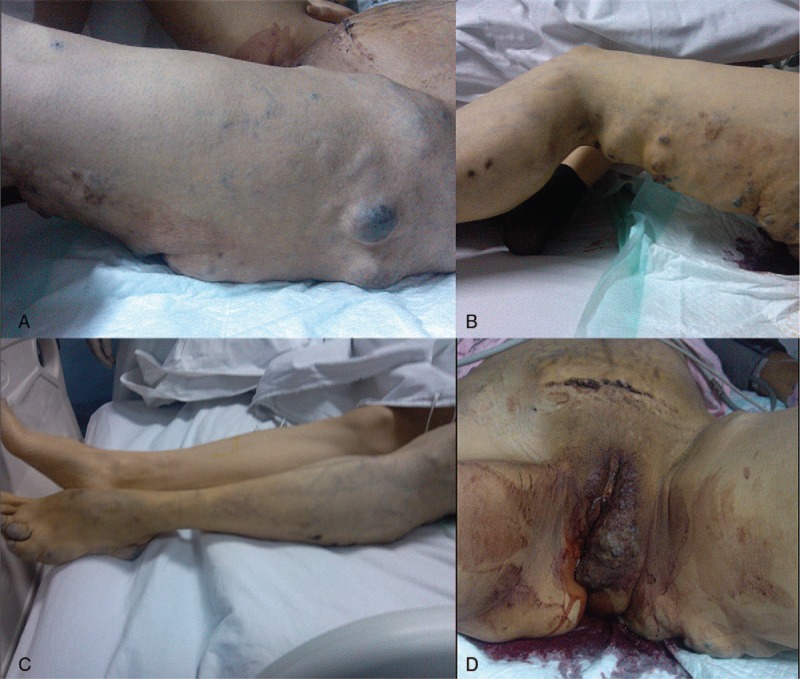
(A–C) Multiple subcutaneous hemangiomas, varicosities and port-wine capillaries distributed over the left buttock and the whole left lower extremity. (D) The left labia majora contained a large complex of varicosities.

At the time of admission, physical examination was remarkable for blood pressure of 89/56 mm Hg, heart rate of 125 per minute, respiratory rate of 22 per minute, and a temperature of 37.3°C. She appeared pale and diaphoretic. Cardiopulmonary examination was unremarkable. The lower abdomen was bulging and tender, with scattered subcutaneous ecchymoses. The bottom of the uterus was palpable at the level of navel with tenderness. A Pfannenstiel incision scar with old blood scab was seen. And there was a palpable mass of 16 × 18 cm around the incision. Multiple subcutaneous hemangiomas, varicosities and port-wine capillaries distributed over the left buttock and the whole left lower extremity (Fig. 1A–C). No bruit or thrill was present. Massive hypertrophy of the left lower extremity was obvious, and the difference in leg length was 4 cm. The left labia majora contained a large complex of varicosities (Fig. 1D). Bimanual examination showed massive bleeding from the vagina without blood clot.

Laboratory studies showed white blood cell (WBC) 9.01 × 10^9^/L (normal 3.69–9.16 × 10^9^/L), hemoglobin (HGB) 75 g/L (normal 113–151 g/L), hematocrit (HCT) 23.6% (normal 33.5–45.2%), mean corpuscular volume (MCV) 91.3 fL (normal 82.6–99.1 fL), platelets (PLT) 91 × 10^9^/L (normal 101–320 × 10^9^/L), prothrombin time (PT) 14.4 seconds (normal 10.4–12.6 seconds), activated partial thromboplastin time (APTT) 37.7 seconds (normal 21.1–36.5 seconds), plasma fibrinogen level (FIB) 0.5 g/L (normal 1.8–3.5 g/L), plasma fibrinogen degradation product (P-FDP) 386.3 Ug/mL (normal 0.0–5.0 Ug/mL), INR 1.24 (normal 0.96–1.16), and d-dimer (normal 0.0–0.55 mg/L FEU) could not be measured out for several times. The ultrasound showed that there was a mixed echo of 10.5 cm × 5.1 cm in the uterine cavity with a dark liquid area of 3.8 × 1.8 cm. The spleen was enlarged with an intercostals’ thickness of 4.6 cm. Ultrasound of the liver, gallbladder, pancreas and the kidneys, etc. revealed unremarkable. Cardiovascular ultrasound showed no evidence of cardiomegaly, abnormal cardiac structure, or arteriovenous fistula of the left lower extremity.

Based on these findings, the patient was first diagnosed as: late puerperal hemorrhage, KTS, DIC, and hemorrhagic shock. Despite the blood transfusion, the massaging of the uterus as well as the use of hemostatics, there was still heavy vaginal bleeding of 1700 mL since admission. And the second laboratory tests showed HGB 70 g/L, PLT 78 × 10^9^/L, PT 13.4 seconds, APTT 38.9 seconds, FIB 0.5 g/L, P-FDP 330.3 Ug/mL. d-Dimer could not be measured out either. And then emergency hysterectomy was performed. There was abdominal incision hematoma of about 250 g, and pelvic hematocele of 900 mL. The inert uterus looked like a big pocket. There was nothing special about the uterine vessels and the bilateral adnexa. Old blood clot of about 500 g was found in the cavity of the uterus. The postoperative course was complicated with *intraperitoneal hemorrhage*, disseminated intravascular coagulation (DIC), peritonitis and sepsis. Thirty-nine hours after the hysterectomy, the peritoneal drainage was bloody about 1800 mL and the HGB dived to 46 g/L with a heart rate of 160 per minute. So, she underwent another emergency laparotomy because of the hemoperitoneum. There was diffused blood oozing in the pelvic and the bleeding site could not be clearly found. So, four conjoint long ribbon gauzes were packed in the pelvic before abdominal closure with the terminal outside. The gauzes were removed, respectively, 36 and 48 hours after the second laparotomy. Then, bilateral internal iliac artery embolization was carried out with the consideration of KTS and the risk of bleeding again. The angiography showed the hypervascularity of bilateral internal iliac artery branches with patchy exudation. The bilateral internal iliac artery was embolized successfully. The *intraperitoneal hemorrhage* was controlled gradually. Meantime, the correction of coagulation disorders, the improvement of anemia, and the anti-infection treatment, etc was carried out. Then, she was sent to the operation room again for wound dehiscence closure. Given her refractory coagulopathy, a diagnosis of Kasabach–Merritt syndrome was considered. Low molecular weight heparin therapy (2000 IU IH qd for 2 days, then 2000 IU IH q12 hour for 2 days, then 3000 IU IH q12 hour for 2 days, followed by 4000 IU IH q12 h) was started at the 13th day after the second laparotomy. And warfarin (2.5 mg orally qn for 3 days followed by 3.75 mg orally qn) was added at the 31st day. The therapy was continued until the 37th day after the second laparotomy, when the patient's hemoglobin and hematocrit were stable and her thrombocytopenia was resolved. Totally, she required transfusion of a total of 43 units of red cell suspension, 9850 mL of fresh frozen plasma, 9 therapeutic doses of platelets, 94 units of cryoprecipitate, 5.5 g lyophilized human fibrinogen, and 300 IU of human prothrombin complex. She was discharged after 42 days of hospitalization with medical elastic stockings. She was followed up for nearly 5 years with nothing remarkable and her baby was healthy.

## 3 Discussion

KTS is a vascular malformation involving capillary, lymphatic, and venous channels. It was diagnosed on the basis of three clinical features usually in one affected extremity: a cutaneous port-wine stain, varicosities, and tissue hypertrophy. The manifestations of this syndrome appear at birth or shortly thereafter. The diagnosis of KTS is based on physical findings and imaging is not needed to establish the diagnosis.^[2]^ However, it should be distinguished from Parkes–Weber syndrome (PWS), a mixed, high-flow, high-shunt arteriovenous malformation, since clinical features, management and prognosis of these two entities are distinctly different.^[3]^ The complications of KTS include haematuria, gastrointestinal bleeding, deep vein thrombosis, thrombophlebitis, and pulmonary embolism, etc and some of them may be life-threatening.^[1,4]^

The etiology of KTS is still obscure. Sreenivasan et al suggested a rare association of KTS and gestational trophoblastic neoplasm with the possible role of hyper-glycosylated Human Chorionic Gonadotropin (HCG) which could stimulate angiogenesis.^[5]^ There are also other observations which suggested a genetic contribution to the occurrence of KTS.^[6–10]^ However, there is no family history in our case. Ultrasound can help diagnose KTS of the fetus with negative family history at 17 and 18 weeks of gestation.^[11,12]^

Genital lesions do not usually cause problems in women who are not pregnant. However, the physiological changes associated with pregnancy may exacerbate the problems of KTS, particularly the risks of hemorrhage and thromboembolism. Both cesarean section and vaginal delivery can be complicated with massive haemorrhage, either due to vascular malformations or due to coagulopathy. Pregnancy and the postpartum period are hypercoagulable status with increased risk of thromboembolism. The administration of anticoagulation therapy in patients with KTS is very important because, in addition to being in a hypercoagulable state, patients with KTS may have coagulation defects that predispose them to bleeding due to the consumption of coagulation factors in the abnormal venous.^[13]^ Kasabach–Merrit syndrome, which has been found in association with hemangiomas, is defined by the presence of thrombocytopenia and the consumption coagulopathy.^[14,15]^ It can occur both on the basis of endothelial damage and on the basis of thrombosis in association with hemangiomas. Massive postpartum hemorrhage and coagulopathy brings the patient into a vicious circle, as described in our case. It has been suggested that patients with either an identified thrombophilic disorder or a prior history of thromboembolic disease in the non-pregnant state should be prophylactically anticoagulated with low molecular weight heparin during the pregnancy and postpartum period.^[16,17]^ Güngor and Jacquemyn have reported two pregnancies with KTS. One of them has not been given anticoagulant therapy during pregnancy and she developed thrombosis after delivery.^[18]^ It seems that our doctors are less experienced in dealing with this kind of complication. There would be a different outcome if the patient in our case was given prophylactic anticoagulation during her pregnancy period or immediately after delivery.

Color Doppler has an important role in following up the vascular malformations and is helpful to make a delivery plan for obstetricians. There is no indication for cesarean section, except for the usual obstetric indication. If neuraxial vascular anomalies cannot be ruled out radiographically, regional anesthesia should be avoided.^[19]^

An interdisciplinary cooperation of obstetrician, anesthesiologist, vascular surgeon and intensive care physician is highly recommended. Thoughtful pre-conceptional counseling, along with systematic antepartum, intrapartum and postpartum care are keys to reduce its mortality and morbidity. Prophylactic anticoagulation is generally advised in the gestational and postpartum period.

## Acknowledgments

We are thankful to the patient for her consent to the report. In addition, we would like to extend special thanks to Lucas Green (medical student, Sichuan University, with his permission) for his help in written English.

## Author contributions

**Conceptualization:** Jian Zhang, Kana Wang.

**Data curation:** Jian Zhang.

**Investigation:** Jian Zhang.

**Writing – original draft:** Jian Zhang.

**Writing – review & editing:** Jian Zhang, Kana Wang, Jie Mei.

Jian Zhang: 0000-0003-4751-3383
